# Coronary sinus flow reserve in response to cold pressor stress in healthy women using velocity-encoded cine (VEC) spiral 3 T MRI

**DOI:** 10.1186/1532-429X-11-S1-P39

**Published:** 2009-01-28

**Authors:** Christopher D Maroules, Alice Y Chang, Andrew Kontak, Hardik Yadav, Tommy Tillery, Ron M Peshock

**Affiliations:** UT Southwestern Medical Center, Dallas, TX USA

**Keywords:** Myocardial Blood Flow, Coronary Sinus, Obstructive Coronary Artery Disease, Cold Pressor Test, Hypertensive Heart Disease

## Introduction

Despite the overwhelming focus on obstructive coronary artery disease over the last few decades, considerable cardiovascular morbidity and mortality is caused by diffuse, non-obstructive disease for which traditional cardiovascular imaging techniques are not suitable. Novel noninvasive techniques that can detect changes in global myocardial perfusion and function might improve the clinical management of patients with diffuse cardiac disease. Coronary sinus flow imaging by velocity-encoding cine MRI has previously been described as a useful measure of global left ventricular perfusion in patients with heart failure, hypertensive heart disease, and cardiac transplants. To our knowledge, this technique has not been investigated using a spiral 3 T MRI technique, which could potentially enhance vessel wall delineation and accelerate scan time.

Prior studies have demonstrated an increase in coronary sinus flow in response to pharmagologic stress. This study tested whether the cold pressor test (CPT), a more specific test of endothelial reactivity could provoke measurable changes in coronary sinus flow.

## Purpose

To determine the feasibility of coronary sinus flow imaging in healthy women in response to the cold pressor test (CPT) using velocity-encoding cine (VEC) spiral 3 T MRI.

## Methods

**Figure 1:**
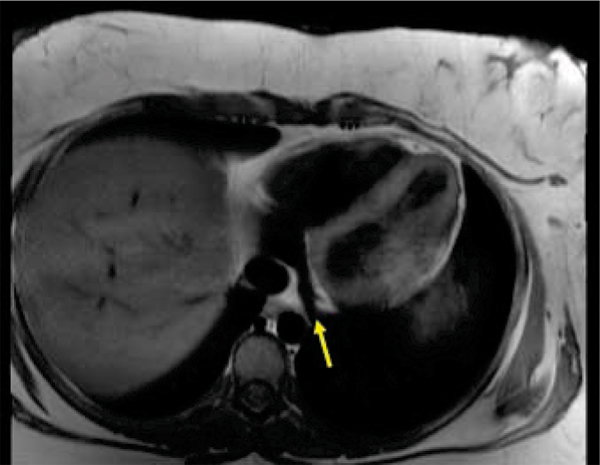
Figure 1

Five healthy women 29 ± 9 years old were enrolled in this study. MR imaging was performed using a 3 T clinical MR scanner (Achieva, Philips, Netherlands) using a 6 element cardiac receiver coil and ECG-gating. Scout images were obtained in the axial plane to localize the coronary sinus (Figure 1). Oblique coronal slices perpendicular to the coronary sinus were aligned 2-cm from the sinus ostium. Velocity-encoded spiral cine sequences were then acquired during end-expiratory breath-holds (8–10 seconds) to measure coronary sinus flow. The following imaging parameters were used: FOV, 25 × 25 cm; matrix, 312 × 312; pixel size, 0.8 × 0.8 mm; slice thickness, 7 mm; TR, 34 msec; TE, 3.5 msec; flip angle 20°; temporal resolution, 69 msec; spiral interleaves, 11; VENC, 80 cm/sec. Heart rate and blood pressure were measured every 30 seconds. After baseline flow data acquisition, the participant's left hand was placed in an ice water bath (50% ice, 50% water) for 3 minutes. Repeat flow images were acquired 1-minute following CPT, and again at 2-minutes and 10-minutes. Images were transferred to a remote workstation and analyzed using QFlow (v. 4.1.6, Medis, Leesburg). The contour of the coronary sinus was manually traced on each magnitude image. Identical tracings were applied to the corresponding phase images so coronary sinus flow velocity and volume flow could be calculated. Coronary sinus flow was determined by integrating phasic flow over time. Coronary sinus flow velocity was determined by averaging flow velocity across all cardiac phases. Statistical significance was determined using the paired *t-* test assuming an α-error of 0.05.

## Results

**Figure 2:**
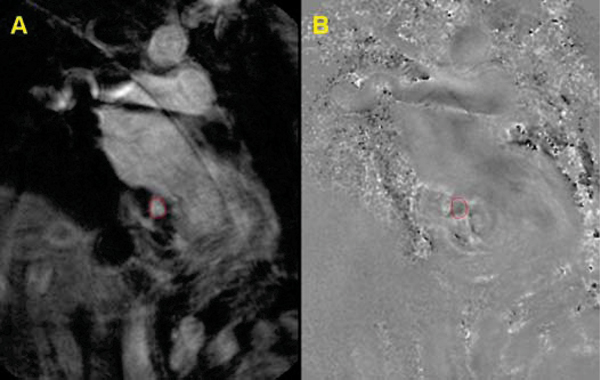
Figure 2

**Figure 3:**
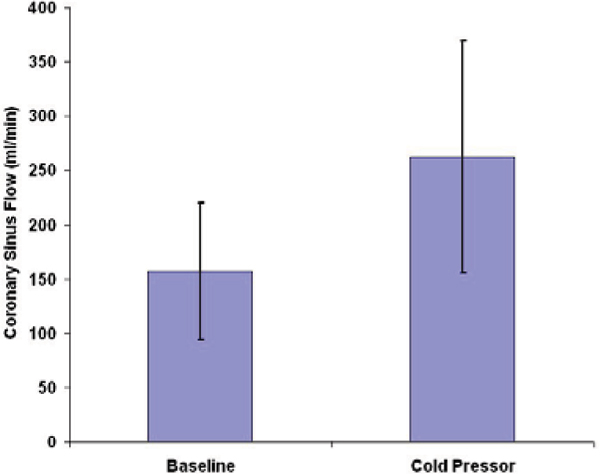
Figure 3

Coronary sinus flow was successfully measured in each subject throughout the cardiac cycle (Figure 2). All subjects tolerated the CPT. Rate-pressure product increased 41.2 ± 15.8% from baseline to peak effect during CPT. Similarly, coronary sinus flow increased from 157 ± 63 ml/minute at baseline to 262 ± 107 ml/minute at peak effect during CPT, representing a 67% increase (*P* < 0.05, Figure 3). Coronary sinus flow velocity increased from 7.9 ± 3.4 cm/second at baseline to 12.6 ± 6.3 cm/second at peak effect during CPT, representing a 61% increase (*P* < 0.05).

## Conclusion

Velocity-encoding cine spiral 3 T MRI is a promising tool for evaluating coronary sinus flow reserve. The CPT provokes a significant increase in myocardial blood flow which can be used to specifically evaluate endothelial function. Future studies will examine the ability of this technique to detect meaningful changes in myocardial flow reserve.

